# Focal Seizures Induced by Intracranial Electroencephalogram Grids

**DOI:** 10.7759/cureus.831

**Published:** 2016-10-15

**Authors:** Eric Hudgins, Mesha-Gay Brown, Brian Litt, Kathryn Davis, Andrew G Richardson, Timothy Lucas

**Affiliations:** 1 Department of Neurosurgery, The Hospital of the University of Pennsylvania; 2 Department of Neurology, University of Colorado Hospital; 3 Department of Neurology, The Hospital of the University of Pennsylvania

**Keywords:** eeg analysis, seizures, refractory epilepsy, epilepsy surgery

## Abstract

Here we present a unique, but important seizure variant directly related to placement of subdural grids. Two distinct epileptogenic zones were identified, one which correlated with the patient’s baseline seizures and a separate zone associated with atypical semiology and localization. Inspection of this zone at surgery revealed cortical deformation from the grid itself. The patient underwent successful surgical resection of the primary epileptogenic zone, but not that of the atypical zone. She remains seizure free at two years following surgery.

Recognition of grid-induced seizures is important as they may confound the interpretation of intracranial electroencephalograms (iEEG) and mislead resective surgery.

## Introduction

The prevalence of epilepsy in developed countries ranges from 0.4-1%, making it a common disabling neurologic disorder [1]. Medically refractory epilepsy occurs in 33% of epilepsy patients, and surgical treatment results in seizure freedom for greater than 50% of these patients, improving quality of life and reducing mortality [2-3]. Intracranial electroencephalograms are indicated when the seizure localization on scalp electroencephalograms (EEGs) is indeterminate or when the clinical, electrographic, and radiographic data are incongruent [4-5]. The spatial resolution and direct cortical contact afforded by iEEG refine seizure location. Subdural electrodes also permit mapping of eloquent cortex prior to resection in cases when the seizure focus is proximate. Successful outcome hinges upon accurate seizure localization and subsequent resection.

Intracranial monitoring is necessarily invasive. The risks associated with iEEG include intracranial hemorrhage, infections, elevated intracranial pressure, cerebrospinal fluid leaks, neurological deficits, and medical complications [5-6]. An under-recognized risk factor may be false location of seizures due to iatrogenic events caused by surgical implants. We discuss such a case and its implications on patient counseling and management. Informed consent was obtained by all participants in this study.

## Case presentation

Here we describe a patient who experienced acute, provoked, right parietal simple partial seizures following the placement of an intracranial grid for mapping of the seizure onset zone. The patient is a 42-year-old right-handed woman with drug-resistant, symptomatic localization-related epilepsy of the right hippocampus, amygdala, and anterior temporal region. She experienced three semiologically different seizure types, two of which were complex partial seizures. Seizure type one was noted as a blank stare and unable to follow commands, followed by episodic memory loss. Seizure type two included oral automatisms of lip smacking and chewing as well as motor automatisms of picking at her clothing with both hands. The third seizure type, further depicted as her “atypical” seizure, was noted only during intracranial monitoring and was characterized by an electric pulse-like sensation through her left hand.

This patient had a history of childhood epilepsy between the ages of 18 months and 17 years. Thereafter anti-epilpetic drugs (AEDs) carbamazepine, phenytoin and phenobarbital were stopped during a seizure-free period of approximately 23 years between 17 and 40 years of age. However, two years prior to hospital admission, the patient began experiencing lapses of time and impaired memory of recent events. Family members noticed that the patient would “zone out” for periods of time and have repetitive mouth movements as well as pick at her clothing. Therefore, typical seizure semiology was characterized by oral and motor automatisms, and memory lapse. These complex partial seizures increased in frequency with an episode occurring every three to four weeks. Levetiracetam was tried at therapeutic doses, but failed to decrease seizure frequency. Lacosamide was then added without a notable decrease in seizure frequency. Seizures were deemed drug resistant.

The patient underwent thorough seizure evaluation. Scalp electroencephalogram during "typical" seizures revealed right mesial temporal onset. Her neuroimaging documented right mesial temporal sclerosis and superior temporal gliosis. To tailor the extent of the lateral resection, she underwent phase II intracranial monitoring.

**Figure 1:**
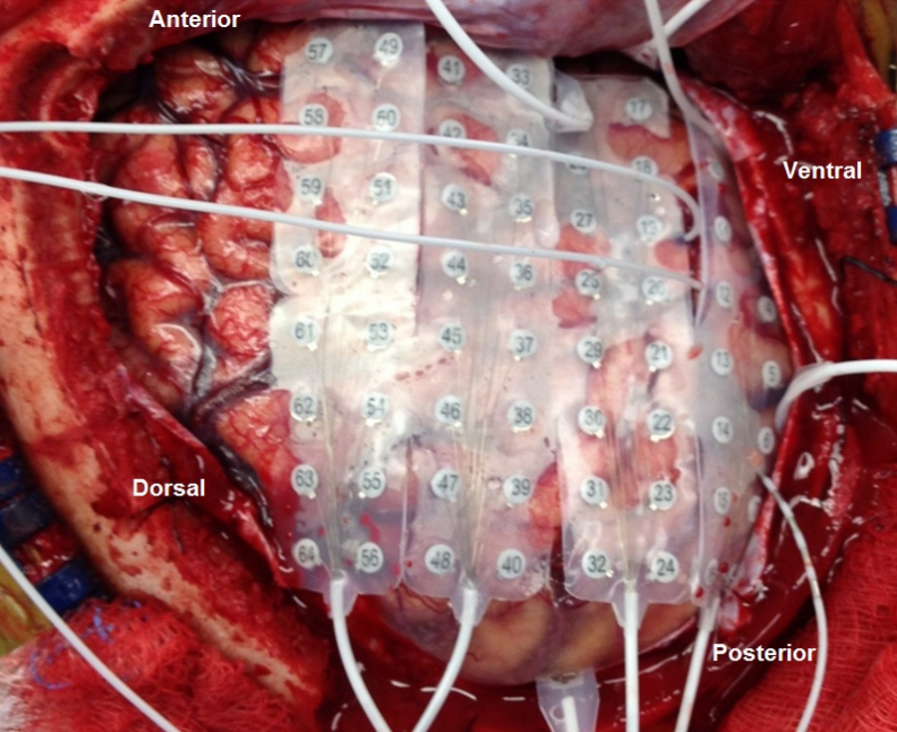
Surgeon's view of iEEG placement Depicted here is the surgeon's view of the right craniotomy immediately following iEEG placement with anatomical locations added for orientation.

A right frontotemporal craniotomy was performed for placement of intracranial electrodes. The implants included a 64-contact grid and a number of strip electrodes and depth electrodes (Figure 1). Strip coverage included the orbitofrontal region, temporal-parietal region, occipital-parietal region, anterior subtemporal, middle subtemporal, and posterior subtemporal regions. The depth electrodes sampled the hippocampal body, amygdala, and superior temporal region.

**Figure 2:**
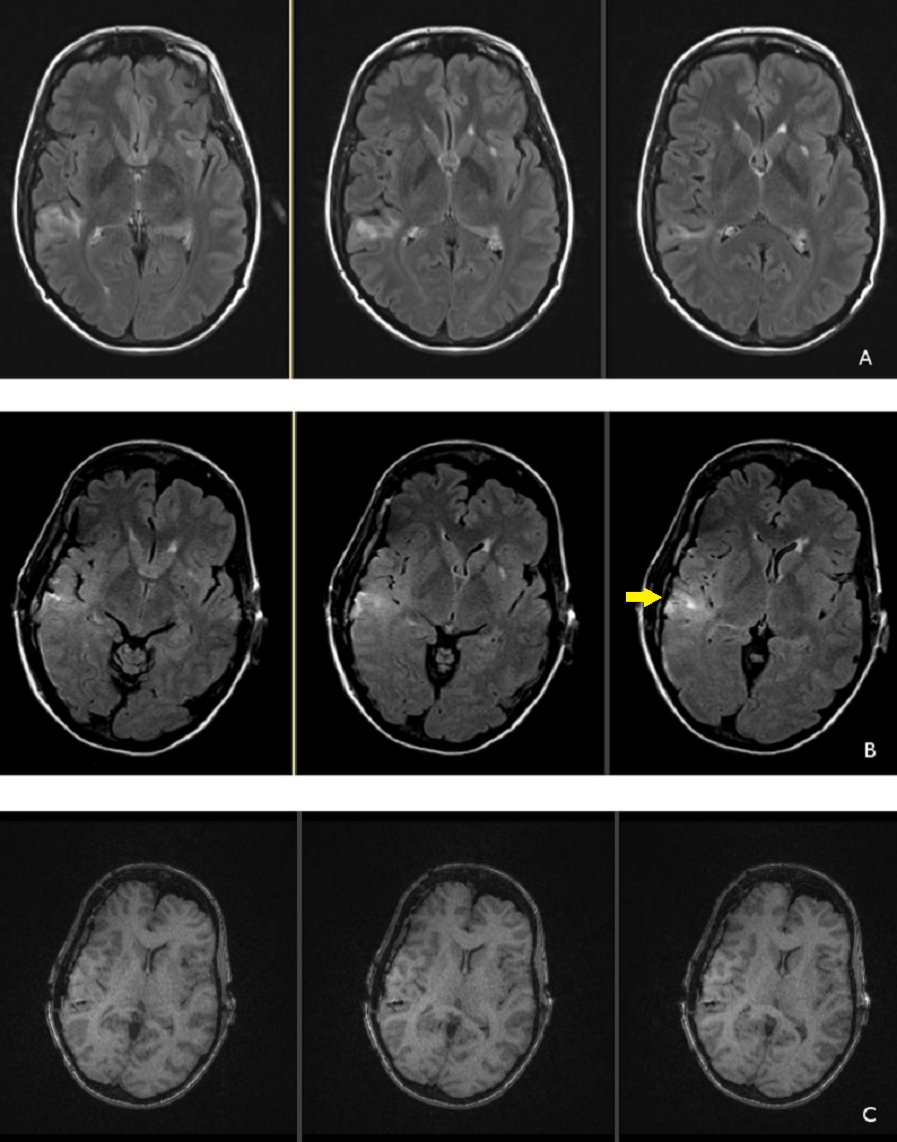
Magnetic resonance images demonstrating postoperative changes Preoperative MRI (A) demonstrating fluid attenuated inversion recovery (FLAIR) hyperintensity in the right posterior parietal area. Postoperative MRI (B) showing similar hyperintensity after grid placement with subtle increase in FLAIR hyperintensity related to grid artifact (arrow). No evidence of cortical contusion is noted. Postoperative changes, T1 non-contrast sequences (C) showing absence of postoperative intraparenchymal hemorrhage.

The patient awoke at her neurological baseline and was transferred to the epilepsy monitoring unit on postoperative day (POD) one. A postoperative magnetic resonance imaging (MRI) scan documented final lead positions (Figure 2) and verified that no cortical injury had occurred during surgery.

**Figure 3:**
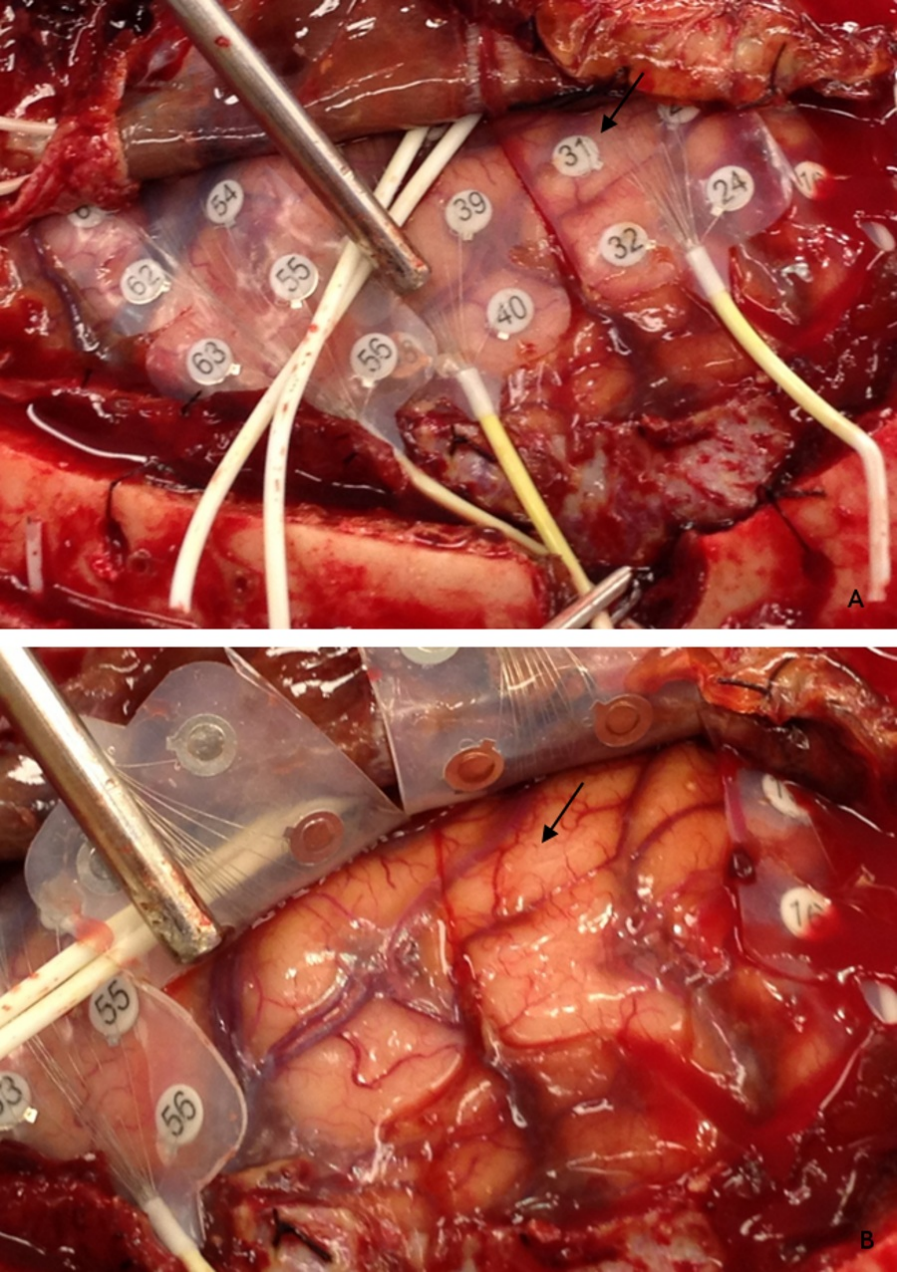
iEEG grid removal (A) Image immediately prior to grid removal showing iEEG grids. Atypical seizures were localized to contact 31 (arrow) and overlaid primary sensory and motor cortex. (B) Cortical impression from iEEG grids near contact 31 (arrow).

**Figure 4:**
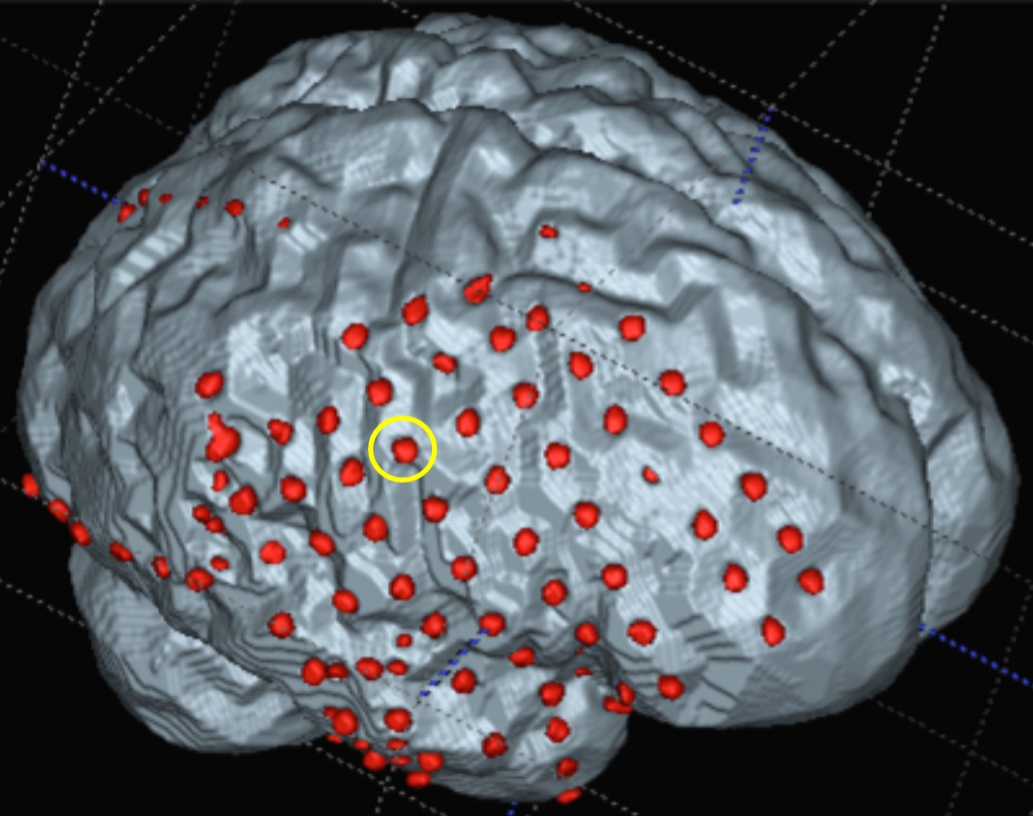
3-D iEEG grid representation Grids superimposed on a three-dimensional magnetic resonance-computed tomography (MR-CT) co-registered surface mask reconstruction of the patient’s cortex. Contact 31 is circled in yellow.

**Figure 5:**
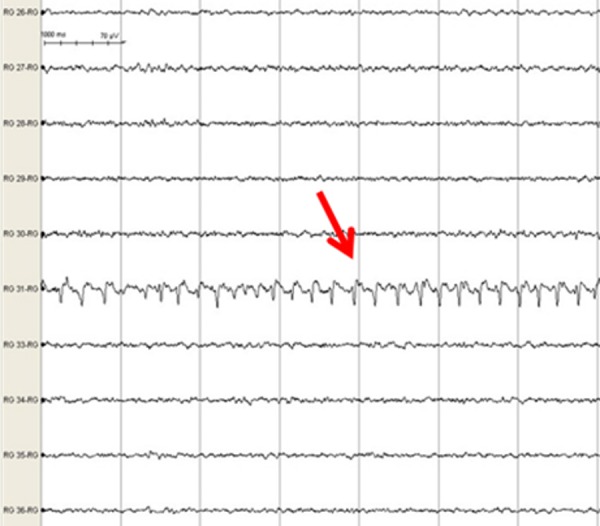
iEEG grid-induced electrographic seizure A seven-second epoch of a 45-second electrographic seizure detected at lead 31. Red arrow points to rhythmic 4 Hz spike-wave activity as seizure evolves.

Frequent, brief, atypical simple partial sensory seizures began on POD two lasting through POD five and were described by the patient as an "electrical pulse" going through her left hand. These seizures had an electrographic onset localized to grid contact 31 (Figures 3-4) and exhibited an abrupt onset of rhythmic 3-4 Hz delta activity followed by evolution to 1-2 Hz delta activity before terminating after two to five minutes (Figure 5). At times there was spread to the adjacent electrodes 23 and 15 without secondary generalization. Greater than 50 atypical seizure episodes occurred with increasing frequency until the patient was loaded with phenobarbitol on POD three in an attempt to control these atypical seizures. These lasted for a total of four days.

The patient experienced four typical clinical and electrographic seizures in the first four days of monitoring. As described earlier, typical seizure semiology consisted of oral and motor automatisms, and memory lapse. All were localized to the right hippocampus (3-4 Hz slowing) with rapid spread to the right amygdala and anterior temporal lobe characterized by rhythmic 5-6 Hz activity. The patient underwent right temporal lobectomy and amygdalohippocampectomy. At surgery, the area around grid contact 31 was carefully inspected. Cortical imprinting in the contour of the grid edges was observed in the immediate vicinity. This was photographically documented (Figure 3). Upon grid elevation, the grid left a 2-4 mm footprint impression which slowly re-expanded during the course of the operation. Following grid removal, all areas of the cortex were visually inspected without evidence of cortical indentation except near grid contact 31. The patient did well postoperatively and remained seizure free at her two-year clinic follow-up appointment. At no point following grid removal did the patient experience atypical seizure events.

## Discussion

We present a case of grid-induced seizures during phase II monitoring. Iatrogenic seizures may confound interpretation of iEEG during seizure localization and adversely affect AED management during monitoring. Early recognition of atypical events following implant and careful correlation of scalp EEG and iEEG may provide clarity.

One possible explanation for the atypical seizures experienced by the patient in this case report is cortical irritation from the grid implantation. This is supported by the local cortical deformation of the brain seen on grid removal and the close temporal association between the new onset of atypical events and surgery. The absence of cortical damage observed on the brain surface, or underlying damage seen on the post-implant MRI, make direct network injury less likely. Grid-induced seizures may prove problematic for future brain-computer interface applications of chronic electrocorticography arrays [7].

Iatrogenic seizures have been previously described with penetrating depth electrodes, but little is known about iatrogenic seizures due to mechanical irritation of surface cortical electrode arrays. While depth electrodes are thought to induce seizures from subclinical vasogenic edema, the etiology of seizures related to subdural surface arrays remains unclear [8]. Potential mechanisms include direct mechanical irritation of cortex, altered hemodynamic properties related to vessel congestion or compression, cerebral edema, or iEEG-induced inflammatory processes. Since it was noted that the patient’s atypical seizures began immediately after grid implantation and ceased upon removal, we deduce direct mechanical irritation could have played a predominant role. Atypical seizures due to cortical indentation from implanted iEEG arrays are rare in clinical practice. We hypothesize that, in this unique case, the indentation's proximity to the primary sensory cortex could potentially explain the occurrence of atypical sensory seizures related to grid placement in this patient (Figure 4). Future work investigating thinner, more flexible materials to construct iEEG arrays may help mitigate the risk of grid-induced seizures.

## Conclusions

Early recognition of the presence of grid-induced seizures is important for iEEG implanted patients. These seizures may confound the interpretation of typical seizures and suggest alternate seizure foci remote from the primary epileptogenic zone. Equally important is the fact that AED management may be influenced by the occurrence of atypical events. The potential for grid-induced seizures will impact development of brain-machine interfaces involving electrode arrays and promote the design of thinner, flexible materials to minimize cortical irritation.
